# Exosomes Secreted from Induced Pluripotent Stem Cell-Derived Mesenchymal Stem Cells Accelerate Skin Cell Proliferation

**DOI:** 10.3390/ijms19103119

**Published:** 2018-10-11

**Authors:** Soo Kim, Seul Ki Lee, Hyunjung Kim, Tae Min Kim

**Affiliations:** 1 Stem Cell Center, Asan Institute for Life Science, Asan Medical Center, Seoul 05505, Korea; skim@amc.seoul.kr (S.K.); clotilda@hanmail.net (S.K.L.); guswjd2949@naver.com (H.K.); 2 Graduate School of International Agricultural Technology and Institute of Green-Bio Science and Technology, Pyeongchang Daero 1447, Seoul National University, Pyeongchang, Gangwon-do 25354, Korea

**Keywords:** iPSCs, MSCs, exosome, wound healing

## Abstract

Induced pluripotent stem cell (iPSC)-derived mesenchymal stem cells (iMSCs) serve as a unique source for cell therapy. We investigated whether exosomes from iMSCs promote the proliferation of human keratinocytes (HaCaT) and human dermal fibroblasts (HDFs). iPSCs were established from human Wharton’s jelly MSCs and were allowed to differentiate into iMSCs. Exosomes were collected from the culture supernatant of MSCs (MSC-exo) and iMSCs (iMSC-exo), and their characteristics were investigated. Both exosome types possessed basic characteristics of exosomes and were taken up by skin cells in vitro and in vivo. A significant increase in HaCaT proliferation was observed with iMSC-exo, although both exosomes increased the viability and cell cycle progression in HaCaT and HDFs. No significant difference was observed in the closure of wound scratch and the expression of reparative genes between cells treated with the two exosome types. Both exosomes enhanced the secretion of collagen in HaCaT and HDFs; however, an increase in fibronectin level was observed only in HaCaT, and this effect was better with iMSC-exo treatment. Only iMSC-exo increased the phosphorylation of extracellular signal-regulated kinase (ERK)-1/2. Our results indicate that iMSC-exo promote the proliferation of skin cells by stimulating ERK1/2 and highlight the application of iMSCs for producing exosomes.

## 1. Introduction

Skin wound healing is characterized by continuous tissue regeneration orchestrated by multiple cells in the epidermal and dermal layers to re-establish the barrier [1]. Upon injury, keratinocytes migrate from the basal layer to the wound area and proliferate for the restoration of the epidermal layer. Fibroblasts also move to the wound site in a spatio-temporal manner and release soluble factors such as growth factors and cytokines and produce the extracellular matrix (ECM) to reconstitute the dermal layer [2]. Despite this well-coordinated response against damage signals, the natural processes of re-epithelialization and dermal repair may be insufficient in severe pathological conditions such as burns, trauma, and diabetes, which necessitate the development of alternative therapeutic strategies such as the use of mesenchymal stem cells (MSCs) and induced pluripotent stem cells (iPSCs) [3].

Mesenchymal stem cells are progenitor cells of connective tissues (osteoblasts, chondrocytes, and adipocytes) with significant immunoregulatory and regenerative functions [4]. Despite their potential therapeutic applications, current technology faces several hurdles such as low viability of the transplanted cells, innate heterogenicity, unidentified factors related to aging of the donor, and potential tumorigenicity [5]. In this regard, the utilization of extracellular vesicles (EVs) secreted by MSCs is recognized as an alternative to overcome the potential risks that may arise from MSCs for clinical uses [6]. EVs are natural nanoparticles enclosed by a lipid bilayer that are released in response to the microenvironment. EVs deliver their membrane signals or internal contents of parental cells, including nucleic acids, lipids, mitochondria, and proteins, into the target cell, resulting in the physiological changes in the target [7,8]. Among EV subpopulation, exosome is gaining increasing interests in immune modulation and regenerative therapy [6,8,9].

Induced pluripotent stem cells hold tremendous advantages for tissue regeneration, owing to the high proliferative and differentiation potentials. Furthermore, iPSCs are immunologically safe in autologous transplantation [10,11]. iPSCs may be converted into most cell types, including MSC (induced MSC; iMSC) in a controlled, scalable manner to obtain sufficient number of cells [12]. Several preclinical experiments have shown that iMSCs contribute to the enhancement in the regeneration of several tissues, including blood vessel, periodontal tissue, liver, heart, chondrocyte, skeletal muscle, and cutaneous wound [13,14,15,16,17,18,19].

Despite the potential advantages of utilizing exosomes from iMSCs [6,12], however, it is not clear whether they can stimulate skin cell growth in a similar manner to those derived from MSCs. Thus, we conducted a comparative study on the ability of exosomes from MSCs (MSC-exo) and iMSCs (iMSC-exo) in promoting skin cell proliferation. Our findings demonstrate that exosome from iMSCs (iMSC-exo) exert superior functions as compared with exosomes derived from naïve MSCs (MSC-exo), which is suggestive of their potential application for cell-free skin regeneration strategies.

## 2. Results

### 2.1. Characterization of iMSCs

**Figure 1:**
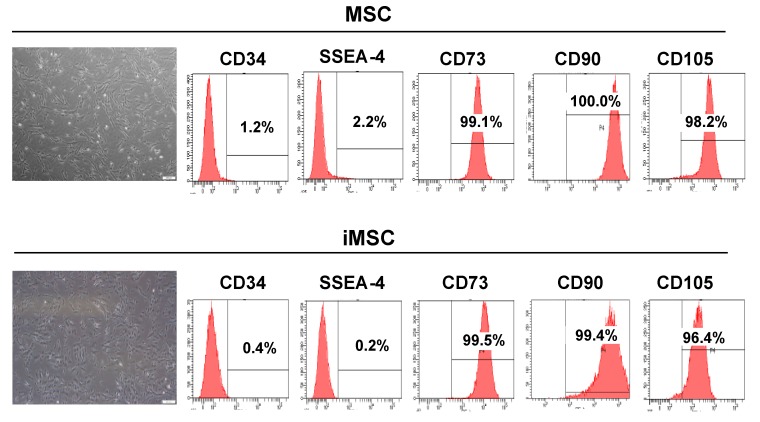
Comparison of the morphology and cell surface marker profile between human Wharton’s jelly MSCs and iMSCs. Both types of cells showed a typical morphology of MSC, with spindle- or fibroblast-like appearance. Flow cytometry analysis showed that both types of cells were positive for MSC markers CD73, CD90, and CD105, but negative for CD34 and SSEA-4. Scale bars are 200 µm.

**Figure A1:**
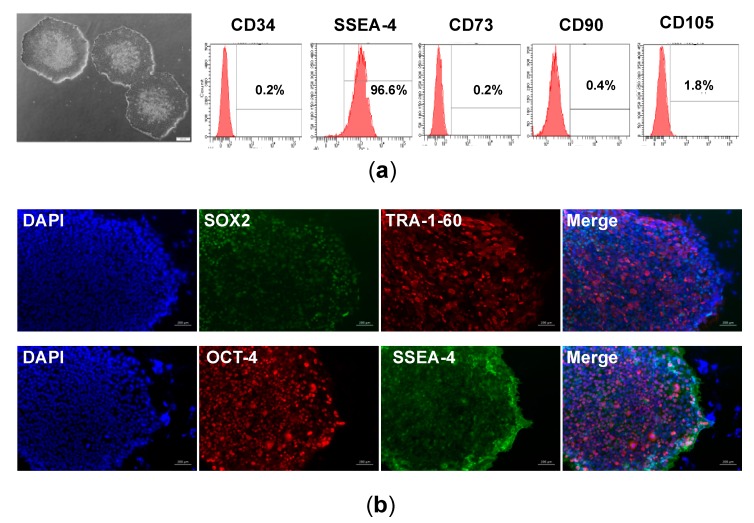
Characterization of iPSCs. (**a**) Light microscopic image and flow cytometry analysis of iPSCs. iPSCs were negative with typical MSC markers (CD73, CD90 and CD105) and endothelial/hematopoietic marker (CD34), while positive with pluripotency marker SSEA-4. (**b**) Immunofluorescence analysis of iPSCs with pluripotency markers. iPSCs were positive with SOX2, TRA-1-60, OCT-4 and SSEA-4. Scale bars are 200 μm. Nuclei were stained with DAPI for counterstaining.

In this study, we sought to investigate whether exosomes secreted from iMSCs can stimulate the proliferation of keratinocytes and dermal fibroblasts, the essential players in skin regeneration. We first isolated and cultured MSCs from the Wharton’s jelly tissue of neonatal umbilical cord and then reprogrammed these cells into iPSCs (Figure A1). iPSCs were differentiated to iMSCs and a total of three lines of iPSC-iMSCs were used to produce exosomes. Throughout the study, we also included three Wharton’s jelly MSCs to compare their characteristics and functionality. iMSCs showed typical fibroblast-like, spindle-shaped appearance after 20 days of induction (Figure 1), and no morphological difference between MSCs and iMSCs was found. Flow cytometric analysis showed that iMSCs were positive for typical MSC surface markers such as CD73, CD90, and CD105 and negative for CD34 and SSEA-4, which are hematopoietic and pluripotent stem cell markers, respectively.

### 2.2. Characterization of Exosomes

**Figure 2:**
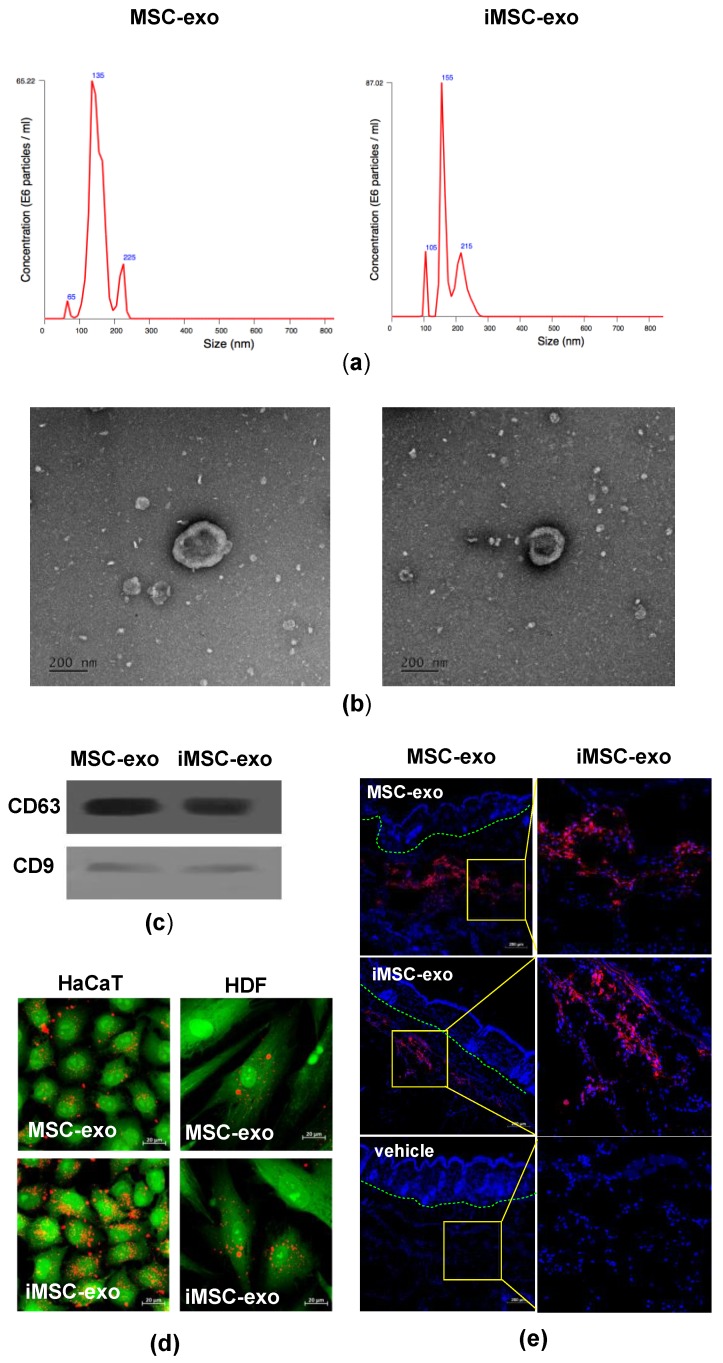
Characterization of exosomes derived from MSCs and iMSCs. (**a**) Nanoparticle analysis of MSC-exo and iMSC-exo. The mean diameter was 167 and 147 nm for MSC-exo and iMSC-exo, respectively. (**b**) TEM analysis of exosomes. Scale bars are 200 nm. (**c**) Immunoblotting for CD63 and CD9 in exosomes. (**d**) Verification of the uptake of exosomes in skin cells. MSC-exo or iMSC-exo (20 µg/mL) were stained with PKH26^®^ (red) and incubated with HaCaT and HDFs for 24 h. Before analysis, cells were counterstained with CellTracker^®^ (green). Scale bars are 20 µm. (**e**) Confocal images of mouse skin tissues treated with MSC-exo or iMSC-exo. A total of 30 µg of PKH26-labeled (red) exosomes were injected into the dorsal skin and tissues were collected after 24 h. Saline (vehicle) was used as negative control. Nuclei were stained with DAPI (blue) for counterstaining. Green dotted lines delineate epidermal-dermal junction. Scale bars are 200 µm.

We collected exosomes from the supernatant of iMSC culture by ultracentrifugation, and their basic characteristics were assessed by comparison with those of exosomes collected from MSCs. We found that the quantity and size distribution of exosomes from two cell types showed no significant difference, as evident from the results of nanoparticle tracking analysis (Figure 2a). Transmission electron microscopy (TEM) analysis showed that the mean diameter of MSC-exo and iMSC-exo was 167 and 147 nm, respectively (Figure 2b). Immunoblot analysis showed that iMSC-exo expressed the exosomal markers CD63 and CD9 (Figure 2c). Co-incubation of human keratinocytes (HaCaT) and human dermal fibroblasts (HDFs) with PKH26^®^-labeled exosomes revealed the uptake of exosomes into the cells (Figure 2d). To see whether these exosomes can be absorbed into skin tissue in vivo, we injected 30 µg of fluorescently labeled exosomes in 1 mL of phosphate-buffered saline (PBS) into mice dorsal skin and found that these were incorporated into cells from the dermal layers after 24 h of treatment (Figure 2e). These results suggest that iMSC-exo have general characteristics of exosomes and may be internalized into skin cells in vivo.

### 2.3. Assessment of the Role of iMSC-Exo in Cell Proliferation and Migration

**Figure 3:**
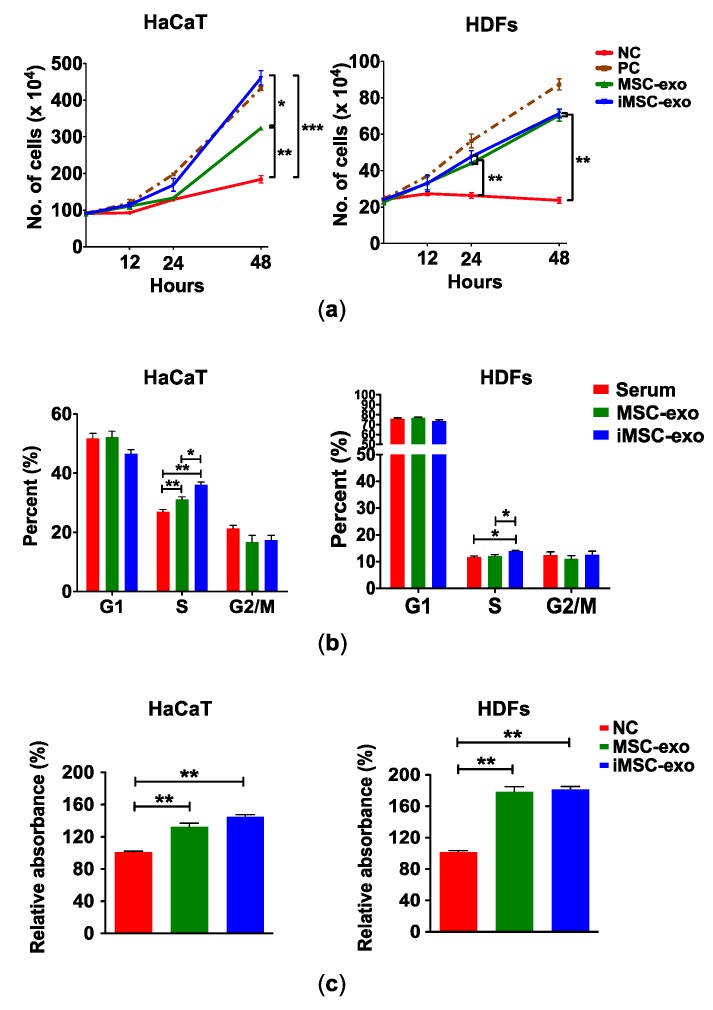
Growth kinetics, cell cycle, and survival analyses of skin cells treated with exosomes. Exosomes collected from MSCs (MSC-exo) or iMSCs (iMSC-exo) were incubated with HaCaT (left) or HDFs (right). (**a**) Growth profile was measured in exosome-treated cells at designated study points. Negative control (NC) is cells from serum-free culture. Culture with serum (10%) was used as positive control (PC). (**b**) At 48 h of treatment, the percentage of cells in each cycle was measured by flow cytometry. Cells cultured in serum (10%) were used as positive control. (**c**) Cell proliferation analysis by MTT assay. At 48 h of exosome treatment, the absorbance of final precipitates was measured at a wavelength of 570nm, and normalized against the value obtained from serum-free negative control (NC). All data are expressed mean ± standard deviation (SD) from three replications. * *p* < 0.05, ** *p* < 0.01, and *** *p* < 0.005.

For functional analysis, we investigated whether iMSC-exo stimulate the growth of HaCaT and HDFs. As shown in Figure 3a, MSC-exo and iMSC-exo stimulated the growth of HaCaT and HDFs. Interestingly, iMSC-exo enhanced the growth rate of HaCaT, as compared with MSC-exo at 48 h (*p* = 0.0104), while no difference was found in the effect of MSC-exo and iMSC-exo on HDFs. Based on these results, we conducted cell cycle analysis to confirm the proliferative role of exosomes. Figure 3b shows that treatment of HaCaT with MSC-exo and iMSC-exo significantly increased the number cells in S phase as compared with cells cultured in serum-supplemented medium (*p* < 0.01). More HaCaT cells were detected in S phase following treatment with iMSC-exo than with MSC-exo (*p* < 0.05). Similarly, iMSC-exo treatment led to an increase in the number of HDFs in S phase, as compared with treatment with serum-supplemented medium or MSC-exo. The results of 3-(4,5-dimethylthiazol-2-yl)-2,5-diphenyltetrazolium bromide (MTT) assay revealed that the treatment of cells with MSC-exo or iMSC-exo resulted in a significant increase in the proliferation of HaCaT and HDFs (Figure 3c).

### 2.4. Wound Scratch Assay

**Figure 4:**
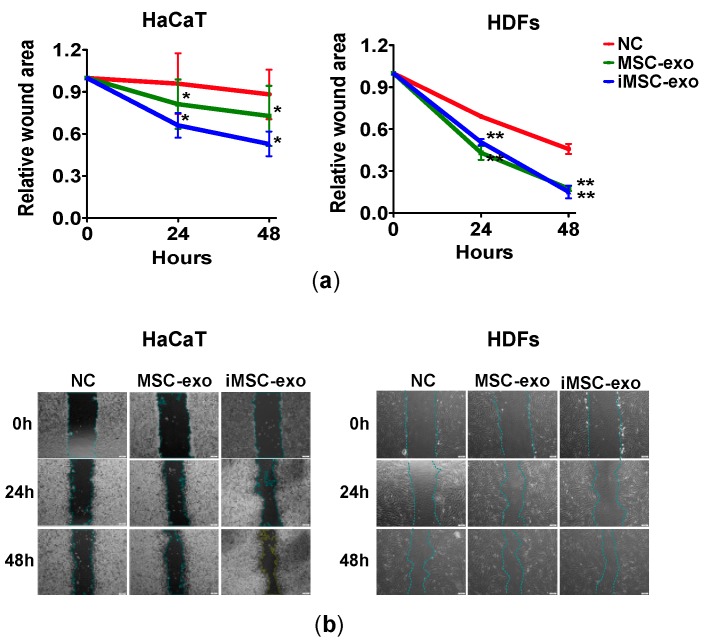
Wound scratch assay of skin cells treated with exosomes. (**a**) Relative wound area changes by exosome treatment. MSC-exo or iMSC-exo were co-incubated with HaCaT (left) or HDFs (right), and the wound area at designated study points was normalized against that obtained at 0 h. NC, negative control (serum-free culture). * *p* < 0.05, ** *p* < 0.01. (**b**) Light microscopy images of wound scratch assay at designated study points. The wound area of HaCaT was calculated using inherent protocol in ImageJ software, while that of the HDFs was manually delineated and subjected to ImageJ software analysis. NC, negative control (serum-free culture). Scale bars are 200 µm.

Wound scratch assay revealed that treatment with both MSC-exo and iMSC-exo significantly reduced the wound area, as compared with negative control (serum-free culture) treatment at 24 and 48 h in HaCaT and HDFs (Figure 4).

### 2.5. Soluble ECM Protein and mRNA Expression Analysis

**Figure 5:**
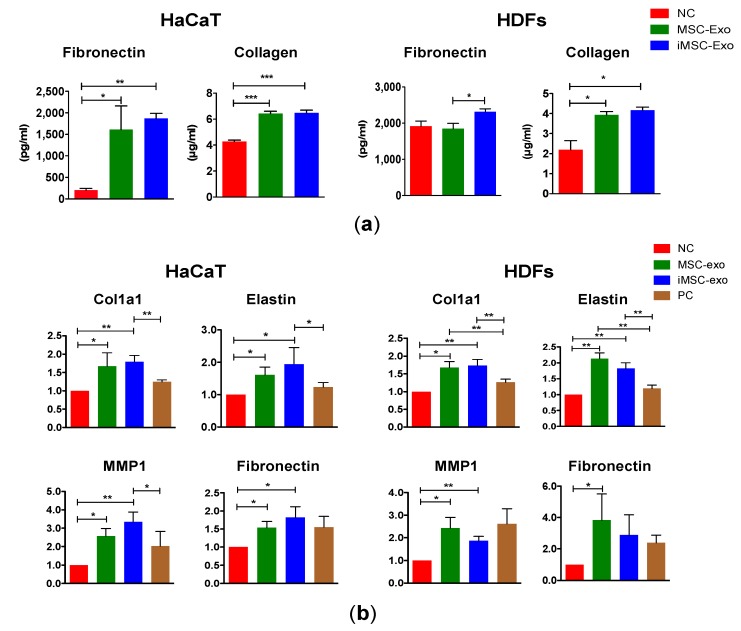
Comparison of the relative soluble protein and mRNA expression following exosome treatment. (**a**) A total of 20 µg/mL of MSC-exo or iMSC-exo were incubated with HaCaT and HDFs for 48 h, and the concentration of fibronectin and collagen was measured using a Human Magnetic Luminex^®^ Screening Assay and SirCol assay kits, respectively. (**b**) Exosome-treated HaCaT and HDFs were subjected to qRT-PCR analysis and the expression of each gene was normalized against the expression detected in the non-treated negative control (NC). Cells cultured with serum (10%) were used as positive control (PC). Negative control (NC) was cells cultured without serum. All data are expressed as mean ± standard deviation (SD) from three replicates. * *p* < 0.05, ** *p* < 0.01, *** *p* < 0.005.

We next determined whether iMSC-exo stimulate the secretion of fibronectin and collagen, which are critical wound healing mediators [20] in HaCaT and HDFs. Figure 5a shows that both MSC-exo and iMSC-exo enhanced the secretion of fibronectin in HaCaT and that this effect was more prominent following treatment with iMSC-exo (*p* < 0.05 and *p* < 0.01 in MSC-exo and iMSC-exo, respectively). We also found a significant increase in collagen secretion in HaCaT, and the effect was similar following MSC-exo and iMSC-exo treatment. In HDFs, treatment with both type of exosomes had no effect on the level of fibronectin, although iMSC-exo were found to be more potent in inducing fibronectin secretion than MSC-exo. An increase in collagen level was observed after MSC-exo or iMSC-exo treatment in HDFs (*p* < 0.05), although no significant difference was found between two exosome types.

To determine whether iMSC-exo regulate the expression of genes that are related to skin wound healing, HaCaT and HDFs treated with iMSC-exo were subjected to quantitative reverse-transcription polymerase chain reaction (qRT-PCR) analysis. As a result, we found that genes involved in wound healing (collagen type 1 α [Col1a1], elastin, and matrix metalloproteinase-1 [MMP1]) [20] showed increased expression after MSC-exo or iMSC-exo treatment in HaCaT and HDFs, as compared with serum-free (negative control) treatment (Figure 5b, *p* < 0.05). The expression of Col1a1, elastin, and MMP1 was higher in HaCaT treated with iMSC-exo than those from the positive control group (*p* < 0.05). In HDFs, the expression of Col1a1 and elastin was higher in cells treated with MSC-exo or iMSC-exo than those from the positive control group, while we failed to observe this effect for MMP1 and fibronectin. No significant difference was detected in the expression of these four genes between cells treated with MSC-exo and iMSC-exo.

### 2.6. Immunoblotting

**Figure 6:**
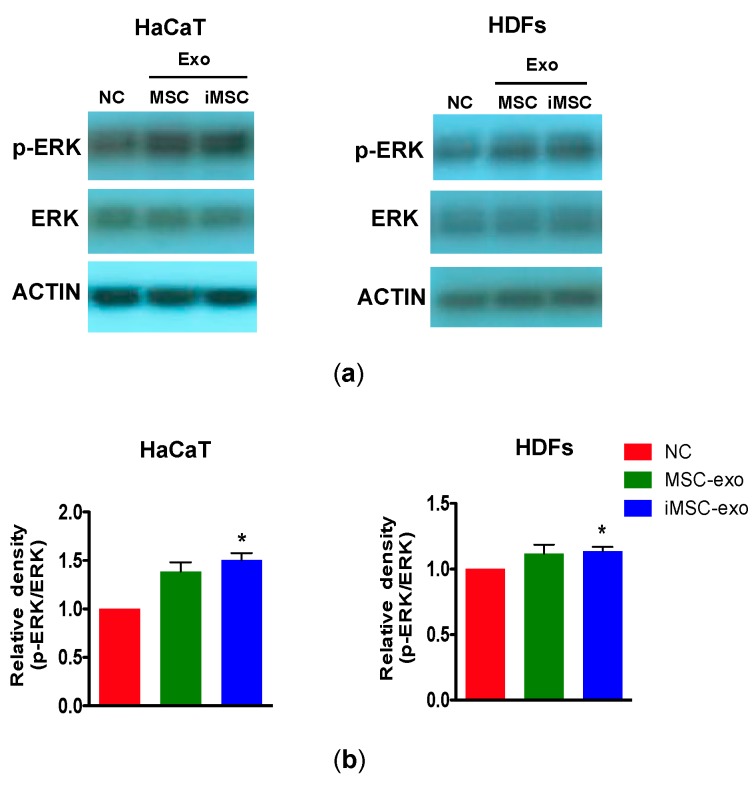
Immunoblotting for the detection of phosphorylated ERK1/2 in HaCaT and HDFs. (**a**) Cells were cultured with MSC-exo or iMSC-exo for 1 h and then the cell extracts were analyzed by immunoblotting for total ERK1/2 and phosphorylated ERK1/2 (Thr202/Tyr204). Beta-actin was used as the loading control. NC and PC are cells cultured without and with serum, respectively. (**b**) Densitometric analysis of the relative level of phosphorylated ERK1/2 (Thr202/Tyr204) against their total levels in HaCaT and HDFs. The value was normalized against that observed for negative control (NC, no serum). All data are expressed as mean ± standard deviation (SD) from three replicates. * *p* < 0.05 against NC. HDFs indicate human postnatal dermal fibroblasts.

We investigated whether the proliferation of skin cells by exosomes derived from MSCs or iMSCs was mediated through the activation of extracellular signal-regulated kinase (ERK)-1/2, which is known to be important for cell proliferation [21]. HaCaT and HDFs were treated with MSC-exo or iMSC-exo and subjected to immunoblot analysis for the detection of phosphorylated and total ERK1/2. As a result, we found that ERK1/2 was activated in HaCaT and HDFs following iMSC-exo treatment as compared with cells cultured without serum (*p* = 0.0195 and *p* = 0.0446 in HaCaT and HDFs, respectively, NC; negative control). On the other hand, MSC-exo treatment failed to induce the phosphorylation of ERK1/2. (Figure 6a,b).

## 3. Discussion

The purpose of this study was to explore whether exosomes derived from iMSCs can exert comparable function as those from MSCs in promoting the proliferation of skin cells. To this end, we isolated and cultured MSCs from the Wharton’s jelly tissue of the neonatal umbilical cord and reprogrammed these cells into iPSCs. iPSCs were subsequently differentiated to iMSCs and used for producing iMSC-exo. In addition, experiments using exosomes from naïve Wharton’s jelly MSCs (iMSC-exo) were conducted. No remarkable difference was detected in their basic MSC characteristics, including morphology and cell surface marker expression. iMSC-exo accelerated the growth, migration, survival, and cell cycle progression of keratinocytes and HDFs. iMSC-exo had better effects than MSC-exo on the proliferation of keratinocytes, and the growth kinetics were similar to those of cells cultured in serum-supplemented medium. The expression of genes involved in wound healing increased following exosome treatment, and the increase in expression was more significant following treatment with iMSC-exo than with MSC-exo. Furthermore, ERK1/2 phosphorylation was increased only after iMSC-exo treatment in both keratinocytes and HDFs. From these findings, we suggest that iMSC-exo have superior (or were at least comparable to MSC-exo) functions than MSC-exo in enhancing the growth and survival of keratinocytes and HDFs.

Current methods for the large-scale preparation of MSCs face several challenges as the amount of MSCs that may be obtained from donors is often insufficient. Furthermore, the potential of growth and differentiation in vitro is affected by various factors such as culture period, donor age, and health condition of donor. Given that iMSCs from iPSCs provide an ideal method that can avoid ethical problem and immune rejection, our strategy for exosome production offers several advantages considering the limitations related to the present applications of MSCs. iPSCs established by an integration-free manner may be indefinitely expanded and readily converted to iMSCs in 20 days. Thus far, only few reports have described the functional comparison between MSC and iMSC. Lian et al. [13] demonstrated that iMSCs obtained from bone marrow-derived iPSCs showed superior functions than native bone marrow-derived MSCs on the recovery of blood perfusion in mouse hindlimb ischemia model, indicating that the function in the ischemic environment differs depending on the cell source. Under hypoxic environment, the levels of stromal cell-derived factor-α and basic fibroblast growth factor were higher in iMSCs, while higher levels of hepatocyte growth factor and nerve growth factor were detected in MSCs. These results suggest that the functional characteristics differ between iMSCs and MSCs, although iMSCs hold basic cellular characteristics of naïve MSCs. In line with these results, our data indicate that exosomes produced from iMSCs and MSCs may have distinct molecular profiles, leading to different effects on cell growth, gene expression, and ERK1/2 phosphorylation. However, studies with animal models of human skin diseases (i.e., burn, wound, baldness, etc.) is needed to corroborate the usefulness of our strategy. Also, determining their molecular profiles, both quantitatively and qualitatively, is needed to assess their potential for clinical application as an alternative to cell therapy.

While MSCs were used for iPSC generation in the present study, it may be ideal to use other somatic cells depending on study purposes. For example, urinary cells would be an optimal for iPSC generation due to the relative easiness of cell acquisition [22]. On the other hand, selecting the optimal cell type for iPSC generation can be a critical issue because the differentiation potential of iPSCs can be affected by the origin of the donor cell [23,24]. We assume that exosomes from iMSCs originated from other somatic cells may have altered potential to those from MSCs used in this study, since the contents of exosomes are known to be changed by the cells from which they were produced. Identifying the biological contents, i.e., transcriptional and epigenetical changes among iMSCs from various donor cells, would provide essential information while determining the donor cell types for different experimental purposes.

Recent studies suggest that cells transmit biomolecules to other cells through the luminal cargo and membrane molecules present in the exosomes [25]. From a translational viewpoint, exosomes secreted from MSCs have shown promising therapeutic effects in a wide range of preclinical models of tissue repair, including kidney, liver, lung, myocardial infarction, cerebral artery occlusion, and skin wound healing [26,27]. Zhang et al. [28] showed that the exosomes from iMSCs accelerated the process of wound repair by enhancing collagen synthesis and vessel formation and through the stimulation of proliferation and migration of HDFs and human umbilical vein endothelial cells (HUVECs). In line with these results, our findings show that these potent roles of iMSC-exo contributed to the activation of ERK1/2 pathways, which have been known to be the main players in cell proliferation [21,29]. The differences in the effects of exosomes from MSC and iMSC on keratinocyte growth may be derived from the distinct pattern of biological cargoes within the exosomes. Thus far, very few attempts have been directed to compare the biological characteristics such as differentiation potential and mRNA profile between naïve MSCs and iMSCs derivatives. Functionally, one recent study demonstrated that iMSCs from fibroblast-derived iPSCs were equivalent to adipose-derived MSCs in reducing the inflammation and lesion of gut in mice model of inflammatory bowel disease (IBD) [30]. On the other hand, Diederichs et al. compared the differentiation ability and gene expression analysis between bone marrow-derived MSCs (BM-MSCs) and autologous BM-MSC-derived iMSCs, and it is found that naïve BM-MSCs have better potential than autologous iMSCs to differentiate into mesenchymal derivatives under osteogenic, chondrogenic and adipogenic conditions [10]. Therefore, iMSCs may be regarded as a distinct population of cells of MSC-like phenotypes derived from the same donor, indicating that the characteristics between MSCs and iMSCs differ in various aspects. The differences in the effects of MSC-exo and iMSC-exo on the proliferation of HaCaT may be associated with the presence of different biological cargos, including surface mitogens or mRNA epigenetics. We found differences in the effects of MSC-exo and iMSC-exo on growth factor signaling pathways. A significant increase in ERK1/2 phosphorylation was detected in keratinocytes and HDFs after iMSC-exo treatment; however, MSC-exo failed to show similar effects. It has been reported that ERK1/2 signaling is activated in HDFs following treatment with exosomes collected from the culture supernatant of BM-MSCs [31]. The reason for the insignificant increase of ERK1/2 phosphorylation by MSC-exo in our study is not clear, but may be attributable to relatively large experimental variation, or different experimental protocols, including the source of exosomes (MSCs from bone marrow versus Wharton’s jelly) and different period of exosome treatment. Overall, more precise methods are warranted, which may quantitate exosomes before testing their functions.

Several studies have demonstrated that stem cell secretome contains various biomolecules that contribute to tissue repair [32,33,34,35,36]. Walter et al. [37] reported that the culture supernatant of MSCs promoted the migration of keratinocytes and HDFs and that ECM components such as collagen types I, V, VI, XII, and fibronectin were present in the MSC-conditioned medium. A recent study using mass spectrometry identified several proteins in the exosomes harvested from acellular fresh Wharton’s jelly tissue that is embedded with MSCs [38]. In particular, these authors demonstrated that Wharton’s jelly exosomes have the capacity to directly contribute to wound healing and that exosomes contain various cargo proteins involved in wound healing, including vimentin, ankyrin, fibrillin, desmin, α-2-macroglobulin, and fibronectin [39]. Therefore, it would be ideal to compare the composition of biomolecules in the exosomes harvested from acellular Wharton’s jelly and culture supernatant of MSCs and iMSCs to identify the underlying mechanism that is responsible for their functions. Lastly, to further understand the mechanism of how exosomes can exert their therapeutic function in vivo, it would also be critical to investigate whether exosomes can activate the endogenous cellular repair mechanism by indirect pathways including increasing the vessel formation in dermal layer [28,40], stimulating the recruitment of other reparative cells (e.g., macrophages), or by enhancing the differentiation of epidermal stem cells in pathological wound healing [41,42].

## 4. Materials and Methods

### 4.1. Isolation and Expansion of MSCs

Wharton’s jelly MSCs were collected from the umbilical cords after a full-term, healthy delivery. The procedures for tissue harvest and obtaining informed consent were approved by the Asan Medical Center Institutional Review Board (protocol no. 2015-3030). Written informed consent was obtained from all participants. The umbilical cords were washed several times with PBS prior to cutting them into small pieces (0.5–1 cm in length). Each piece was then longitudinally cut for the removal of all blood vessels. The matrix was scraped, minced into small pieces, and transferred to 100-mm tissue culture dishes (SPL Life Science, Pocheon-si, Korea). The cells were cultured for 7 days in Minimum Essential Medium-α (MEM-α) (Thermo Fisher Scientific, Waltham, MA, USA) supplemented with 10% fetal bovine serum (FBS) and 1% Pen-Strep (Thermo Fisher Scientific, Waltham, MA, USA) at 37 °C in 5% CO_2_ and 95% humidified air. The culture medium was changed every 4 days. Upon reaching 80–90% confluency, cells were detached with TryPLE Express (Thermo Fisher Scientific, Waltham, MA, USA) and replated into culture flasks at a split ratio of 1:5.

### 4.2. Generation of iPSCs and iMSCs

The MSCs were transduced with CytoTune^®^-iPS 2.0 Sendai reprogramming kit (Thermo Fisher Scientific, Waltham, MA, USA). Medium was changed every other day. At day 7, post-transduction, the transduced cells were transferred onto a vitronectin-coated (Thermo Fisher Scientific, Waltham, MA, USA) six-well plate. The medium was replaced with Essential 8™ medium (Thermo Fisher Scientific, Waltham, MA, USA). The culture medium was changed daily until iPS colonies emerged. Individual colonies were manually picked and transferred to a new vitronectin-coated four-well plate (Thermo Fisher Scientific, Waltham, MA, USA). Established iPSC lines were maintained in Essential 8™ medium at 37 °C with 5% CO_2_ and routinely sub-cultured (1:10) using 50 mM ethylenediaminetetraacetic acid (EDTA)/PBS without Ca^2+^ and Mg^2+^. After 5 days, the iPSC medium (Essential 8™) was replaced with MSC medium (Dulbecco’s modified Eagle’s medium [DMEM] high glucose (Thermo Fisher Scientific, Waltham, MA) supplemented with 15% FBS [ATCC], and 1% antibiotic-antimycotics (Thermo Fisher Scientific, Waltham, MA). The iPSCs were maintained in MSC medium for 2 weeks with the medium being changed every other day. Cells were passaged to gelatin-coated (EMD Millipore, Billerica, MA, USA) tissue culture vessels using TryPLE Express. iMSCs were defined as passage 1 (P1) after the first passage. For maintenance of iMSCs, cells were passaged upon reaching 90% confluency and seeded at a density of 1.6 × 10^4^ cells/cm^2^ to new tissue culture vessels.

### 4.3. Characterization of MSCs and iMSCs

For flow cytometry analysis, MSCs or iMSCs were trypsinized and washed twice prior to resuspension in PBS containing 2% FBS and 1 mM EDTA. Cells were adjusted to 1 × 10^6^ in 100 µL of cell suspension. For cell surface labeling, cell suspensions were incubated at 4 °C for 30 min with 5 µL of antibodies (dilution, 1:20) against MSC and iPSC-specific surface markers. Phycoerythrin (PE)-conjugated mouse anti-human CD73, fluorescein isothiocyanate (FITC)-conjugated mouse anti-human CD90, and PE-conjugated mouse anti-human CD105 antibodies were supplied by BD Biosciences (San Jose, CA, USA). FITC-conjugated mouse anti-human CD34 and PE-conjugated mouse anti-human SSEA4 antibodies were supplied by BD PharMingen™ (San Jose, CA, USA). Cell surface marker analysis was performed using a BD FACSCanto™ II Flow Cytometer and FACSDIVA software version 6.1.3 (BD Biosciences, San Jose, CA, USA).

### 4.4. Collection of Exosomes

Extracellular vesicle-depleted FBS was prepared by filtering with 0.2-μm filter, followed by ultracentrifugation at 100,000× *g* for 18 h at 4 °C. Upon reaching 70% confluency, the culture media from MSCs or iMSCs were replaced with fresh media supplemented with EV-depleted FBS (10%) and the cells were subsequently cultured for additional 48 h. After incubation, MSC and iMSC media were harvested, centrifuged for 5 min at 900× *g*, and the supernatant was centrifuged for an additional 1 h at 10,000× *g*. To remove the particles larger than 200 nm, the supernatants were filtered through 0.2-μm pore filters. Finally, exosomes were isolated by ultracentrifugation at 100,000× *g* for 2 h and the pellet was subsequently washed with PBS and subjected to ultracentrifugation. Exosome pellet resuspended in PBS was evaluated for protein concentration by Bradford assay (Bio-Rad, Hercules, CA, USA). The aliquots were passed through 0.22-μm microcentrifuge filters and stored at −80 °C.

### 4.5. Characterization of Exosomes

The morphology of exosomes was analyzed by TEM. Briefly, samples were applied to glow-discharged carbon-coated copper grids. After allowing the sample to be absorbed for 2 min, the buffer solution was blotted-off onto Whatman paper, and the samples on the grids were stained with 2% (*w*/*v*) uranyl acetate (UrAc) for 1 min. Excess of UrAc was blotted-off. The results were recorded with the Tecnai™ G^2^ Spirit TWIN (FEI Company, Hillsboro, OR, USA) at an acceleration voltage of 120 kV. Nanoparticle analysis was conducted to determine the size and concentration of exosomes using NanoSight 300 (Malvern Instruments, Malvern, UK). The exosomal protein was extracted using Total Exosome RNA and Protein Isolation Kit (Thermo Fisher Scientific, Waltham, MA, USA). The amount of exosomal protein was assessed by the Bradford method.

### 4.6. In Vitro Exosome Uptake Assay

To verify whether the exosomes are internalized into skin cells, cells were cultured overnight at a density of 1 × 10^5^ cells (HaCaT and HDFs) per well in 12-well culture plates. Exosomes from MSCs and iMSCs were labeled with PKH26^®^ red fluorescent cell linker kit (Sigma-Aldrich, St. Louis, MO, USA) according to the manufacturer’s instruction. Labeled exosomes (20 μg/mL) were co-cultured with HaCaT and HDFs for 16 h, and the nuclei were stained with 4′,6-diamidino-2-phenylindole (DAPI). Images were obtained using a Zeiss LSM 780 confocal microscopy system (Carl Zeiss Meditec AG, Jena, Germany).

### 4.7. In Vivo Exosome Uptake Assay

All animal procedures were reviewed and approved by IACUC of Asan Medical Center, Seoul, Korea. C57/BL6 mice (8-week old) were purchased from Orient Bio Inc. (Seongnam, Korea) and maintained in the Laboratory Animal Research Center at Asan Medical Center. At 10 weeks of age, the animals were anesthetized and their backs were shaved as designated in the animal protocol. Subsequently, 100 μL of PBS (vehicle) or MSC-exo or iMSC-exo in PBS was intradermally injected into the back skin using insulin syringe. After 24 h, the skin tissues were harvested, frozen in optimum cutting temperature (OCT) compound (Tissue Tek, Tokyo, Japan), cryosectioned at 10-μm thickness, stained with 10 μg/mL of DAPI, and visualized with fluorescent microscopy (Olympus BX-51, Tokyo, Japan).

### 4.8. Cell Proliferation, Cell Cycle, and Wound Scratch Assay

The proliferation of cells was determined using the standard MTT assay. Cells (1 × 10^4^ cells/well) were overnight cultured in 96-well plates and treated with 10 μL of MTT reagent (R&D system, Minneapolis, MN, USA). A total of 100 μL of purple formazan crystals were dissolved in a detergent reagent and added to the plate. The absorbance was recorded on a microplate reader at a wavelength of 570 nm. For cell cycle analysis, exosome-treated cells were stained with 1 μM of Vybrant™ DyeCycle™ Green according to manufacturer’s instruction and analyzed with a FACS Canto flow cytometer (BD biosciences, Piscataway, NJ, USA). For wound scratch assay, HaCaT and HDFs were cultured overnight at a density of 3 × 10^5^ cells/well in six-well culture plates. Wound was made by scratching using a sterile 1,000 μL pipette tip. After washing the cells with PBS, images were obtained at 0, 24, and 48 h using a phase-contrast microscope. The area of wound was quantified by ImageJ (NIH, Bethesda, MD, USA) and normalized against wound area at 0 h.

### 4.9. Soluble ECM Analysis and Quantitative Real-Time PCR

HaCaT and HDFs cells (3 × 10^5^) were seeded in six-well plates and incubated at 37 °C to allow cell attachment. The cells were treated with exosomes (10 or 20 μg) for 48 h. Cells were harvested and the concentration of fibronectin and collagen in the supernatant was measured using a Human Magnetic Luminex^®^ Screening Assay (R&D Systems, Minneapolis, MN, USA) and SirCol assay kit (Carrickfergus, UK), respectively. For gene expression analysis, total RNA was extracted using TRIzol, and cDNA was synthesized using Power SYBR Green PCR Master Mix (Thermo Fisher Scientific). qRT-PCR was performed using the QuantStudio 6 Flex Real-Time PCR System. The sequences of primers used were as follows: Glyceraldehyde-3-phosphate (GAPDH), 5′-ACATCGCTCAGACACCATG-3′ and 5′-TGTAGTTGAGGTCAATGAAG-3′; elastin, 5′-GGGTTGTGTCACCAGAAAGCA-3′ and 5′-CAACCCCGTAAGTAGGAATGC-5′; Col1A1, 5′-CACAGAGGTTTCAGTGGTTTGG-3′ and 5′- GCACCAGTAGCACCATCATTTC-3′; MMP1, 5′-TTGAGAAAGCCTTCCACTCTG-3′ and 5′-CCGCAACACGATGTAAGTTGTA-3′; and fibronectin, 5′-AAGATTGGAGAGAAGTGGGACC-3′, and 5′-GAGCAAATGGCACCGAGATA-3′. The change in mRNA expression was determined according to the method of 2^−ΔΔ*C*t^ [43].

### 4.10. Immunoblotting

Exosome and cellular proteins were obtained by fractionation of cell lysates with 10% sodium dodecyl sulfate polyacrylamide gel electrophoresis (SDS-PAGE) under reducing conditions. The protein bands were transferred onto a polyvinylidene fluoride membrane and analyzed using rabbit monoclonal anti-CD9 (Abcam, Cambridge, UK), anti-CD63 (Abcam), anti-p44/42 mitogen-activation protein kinase (MAPK; Thr202/204; Cell Signaling, Danvers, MA, USA), polyclonal anti-phospho p44/42 MAPK (Cell Signaling), and anti-β-actin antibodies (Abcam) at 4 °C overnight. Before probing, nonspecific binding was blocked by incubation with 5% bovine serum albumin (BSA) in TBST (10 mM Tris, pH 8.0, 150 mM sodium chloride (NaCl), and 0.5% Tween-20) for 60 min at room temperature. Membranes were washed four times for 10 min each and incubated with horseradish peroxidase-linked goat anti-rabbit secondary antibody (1:3000; Abcam) at room temperature for 1 h. Blots were washed four times with TBST and developed with the enhanced chemiluminescence (ECL) system (Amersham Biosciences, Waltham, MA, USA) according to the manufacturer’s protocols and were quantified by using Image J (Version 1.50, National Institutes of Health, Bethesda, MD, USA).

### 4.11. Statistical Analysis

Differences between two groups were analyzed by Student’s *t*-test using GraphPad Prism 5.0 software. *P* values less than 0.05 were considered as significantly different.

## 5. Conclusions

Our data indicates that iMSC-derived exosomes may promote the growth, proliferation, and migration of skin cells. Considering the limited scalability and immunological problem associated with MSCs, our strategy may have the potential for producing exosomes for therapeutic applications.

## Abbreviations

HDF	Human dermal fibroblast
MSC	Mesenchymal stem cell
iPSC	Induced pluripotent stem cell
iMSC	Induced mesenchymal stem cell
PCR	Polymerase chain reaction

## Appendix A

### Supplementary Methods: Immunofluorescence Analysis of Pluripotency Markers in iPSCs

iPSCs were fixed with 4% paraformaldehyde in PBS for 30 min. Cells were then permeabilized with 0.2% Triton X-100 (Sigma-Aldrich) in PBS for 10 min and subsequently blocked with 5% FBS in PBS for 30 min. All immunostaining was conducted by PSC-4 Marker Immunocytochemistry Kit (Thermo Fisher Scientific, Waltham, MA, USA) according to the manufacturer’s instructions. After last wash, nuclei were counterstained with NucBlue™ Fixed Cell ReadyProbes™, Thermo Fisher Scientific, Waltham, MA, USA) and then incubated for one minute. Images were obtained using a Zeiss LSM 780 confocal microscopy system (Carl Zeiss Meditec AG, Jena, Germany).
